# Supplementary material for: Relationship of mRNA Expression of Selected Genes in Peripheral Blood and Synovial Fluid in Cranial Cruciate Ligament Deficient Stifles of Dogs
Source: Animals (Basel). 2022 Mar 17;12(6):754. doi: 10.3390/ani12060754 (PMC8944536; doi:10.3390/ani12060754)
Supplement: Supplementary file 1 [file animals-12-00754-s001.zip › animals-1541400-supplementary.pdf]

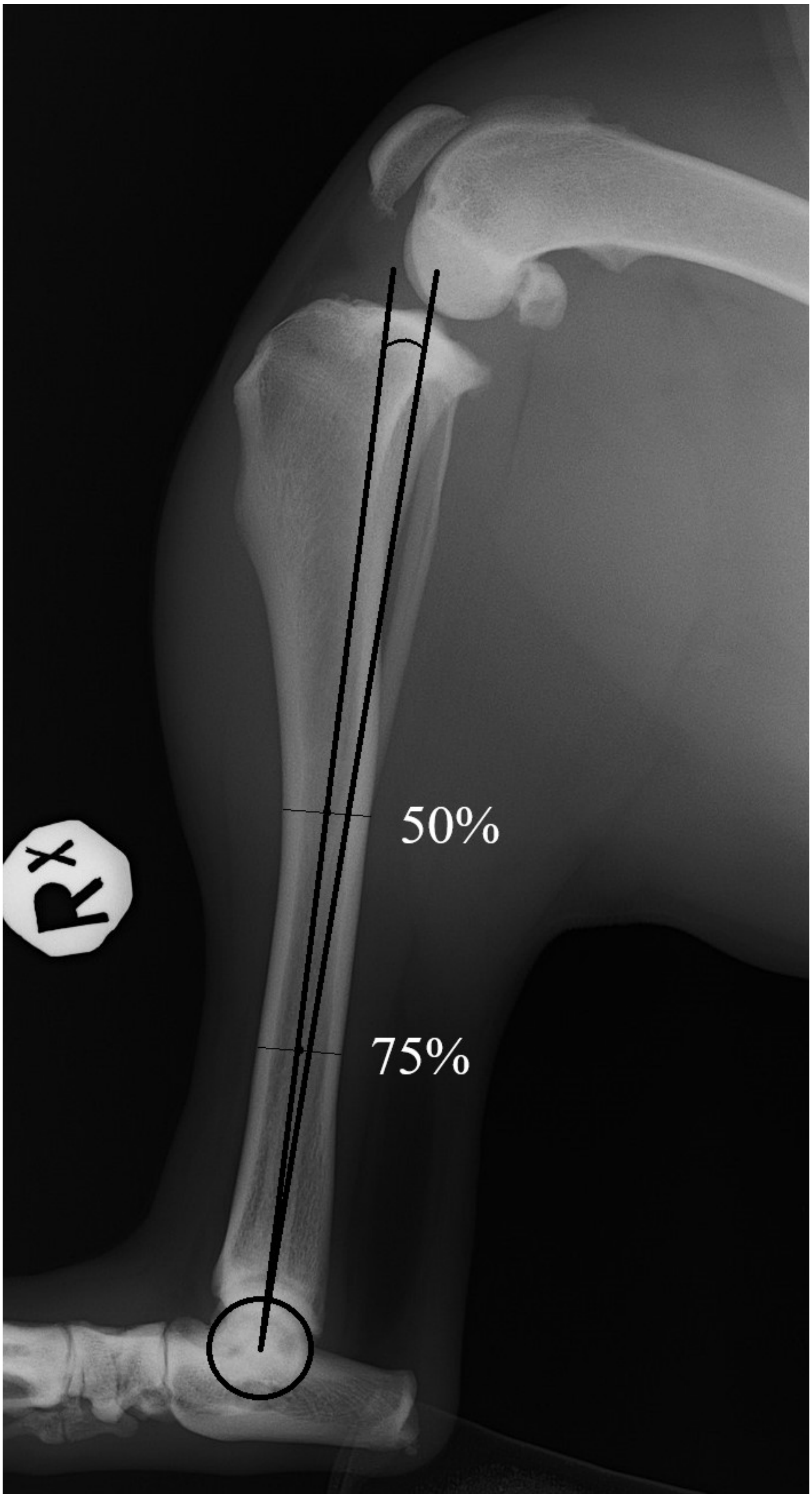

Figure S1 Measurement of AMA-angle. Anatomical axis is equal to connection of midpoint between cranial and caudal cortex at 50% and 75% of the tibial shaft length. The mechanical axis is a line connecting the midpoint of intercondylar

eminence and central point of the tarsal joint. AMA- angle is an angle between the anatomical and mechanical axis.
